# Mg_29–*x*_Pt_4+*y*_: Chemical Bonding Inhomogeneity and Structural Complexity

**DOI:** 10.1021/acs.inorgchem.2c02653

**Published:** 2022-09-27

**Authors:** Laura Agnarelli, Yurii Prots, Reiner Ramlau, Marcus Schmidt, Ulrich Burkhardt, Andreas Leithe-Jasper, Yuri Grin

**Affiliations:** Max-Planck-Institut für Chemische Physik fester Stoffe, 01187 Dresden, Germany

## Abstract

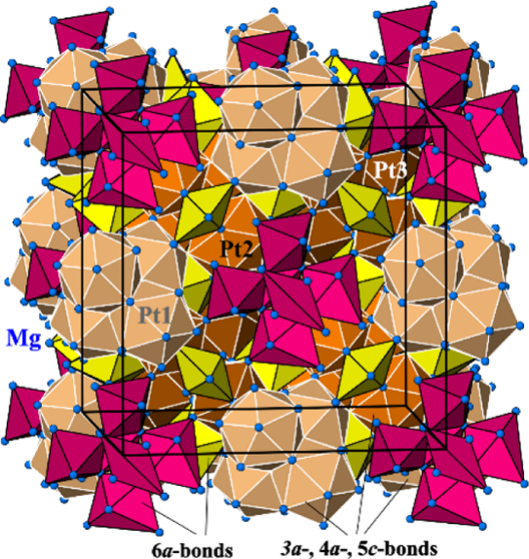

Mg_29–*x*_Pt_4+*y*_ represents the family of complex intermetallic compounds (complex metallic alloys, CMAs). It crystallizes in the cubic non-centrosymmetric space group *F*4̅3*m* with *a* = 20.1068(2) Å and around 400 atoms in a predominantly ordered arrangement. The local disorder around the unit cell origin is experimentally resolved by single-crystal X-ray diffraction in combination with atomic-resolution transmission electron microscopy (TEM, high-angle dark-field scanning TEM) studies. The quantum theory of atoms in molecules-based analysis of atomic charges shows that the unusual mixed Mg/Pt site occupation around the origin results from local charge equilibration in this region of the crystal structure. Chemical bonding analysis reveals for Mg_29–*x*_Pt_4+*y*_—rather unexpected for a crystal structure of this size—space-separated regions of hetero- and homoatomic bonds involving three to six partners (bonding inhomogeneity). Pt-containing 11- and 13-atomic units formed by heteroatomic 3*a*-, 4*a*-, and 5*a*-bonds are condensed via edges and faces to large super-tetrahedrons, which are interlinked by Mg-only 6*a*-bonds. Spatial separation of the regions with different bonding features is the key difference between the title compound and other CMAs, which are characterized by a predominantly homogeneous distribution of heteroatomic bonds.

## Abstract 2

A complex intermetallic compound (CMA) Mg_29−*x*_Pt_4+*y*_ with ca. 400 atoms/unit cell can be described by crystallographic clusters originating from the nested polyhedral units at high symmetry points. From the bonding analysis, the charge transfer contributes strongly to the stabilization of the complex crystal structure. Spatially separated agglomeration of different charges differentiates Mg_29−*x*_Pt_4+*y*_ from a typical CMA with a homogeneous distribution of multi-atomic bonds in the structure.

## 1 Introduction

The chemistry which underlies intermetallic compounds is one of the most complex topics in the field of materials science, mainly because of their broad range of structures, nature of the elements involved, bonding peculiarities, and physical properties.^1−4^ In a previous study, Pauling had already considered the understanding of the bonding mechanisms, which stabilize intermetallic crystal structures, as a special challenge for structural chemistry.^5^ Among this family of inorganic materials, a special category is represented by complex intermetallic compounds (earlier often called complex metallic alloys, CMAs). As their characteristic features were considered giant unit cells, cluster arrangement of atoms with a high number of different atomic environments, where icosahedral coordination plays a prominent role, and inherent disorder—configurational, chemical, or substitutional—with partial site occupancy and split positions.^6−9^ A CMA can show large periodic structures with hundreds or thousands of atoms in the unit cell (like Ta_9063_Al_12827.56_Cu_1244.04_; Pearson symbol: *cF*23134,216^10^) and—at least in the current state of investigations—with a higher frequency of occurrence for binary compounds rather than for ternary compounds.^4,8,9^

A common strategy to describe a CMA from a geometric point of view is the grouping of atoms into local atomic arrangements (crystallographic clusters), which makes the crystal structure easier to visualize, perceive, and understand the geometric organization; it reduces the degree of complexity and allows one to relate the CMA to simpler crystal structures.^6−9,11^ This is the nested polyhedral unit approach,^12,13^ which was introduced first for the description of the γ-brass structure.^14^ The choice of a crystallographic cluster is considered as reasonable, if its shells are as spherical as possible and are quite separate with respect to the distances between the shells and with respect to the center of the nested polyhedrons. On the other hand, the so-defined crystallographic clusters are not in agreement with the chemical definition of such an entity, as the chemical definition entails atomic interactions within the chemical clusters that are stronger than the interaction of the clusters with neighboring aggregates.^15^ Thus, it would be preferable to raise the question about the stabilization mechanisms for these structural units. Based on the intensive studies of crystal structures and electronic configurations of γ-brass phases, the theory of electronic stabilization for metallic materials, known as Hume-Rothery rules, was developed. These rules,^16−18^ which are usually applied for explaining the stability of CMA,^19^ assume a rather homogeneous bonding caused by multi-atomic interactions (metallic bond). Quantum chemical investigations of chemical bonding in CMA are naturally hindered by the huge size of the unit cells and inherent disorder. Thus, the role of the charge transfer in the stabilization was only recently pointed out for an example of the ordered CMA Be_21_Pt_5_^20^ and its chemically analogous but non-complex Be_5_Pt.^21^

Most of the CMA with approx. 400 atoms in the unit cell crystallize in the face-centered cubic Bravais lattice, and—in particular—36 of them crystallize with the space group *F*4̅3*m* (no. 216). Mg-rich transition-metal compounds already reported in the literature^22−30^ can also be considered as CMA since all of them fulfill the conditions mentioned above. In the Mg–Pt system, the possibility of the existence of a Mg-rich phase with the composition Mg_∼6_Pt, similar to that of Mg_6_Pd, was reported already in the 1960s without further details.^24,25,28^ The here-presented joint study of the crystal structure based on single-crystal X-ray diffraction (XRD), together with a transmission electron microscopy (TEM) investigation, and analysis of the chemical bonding with the position-space approach sheds light on the interplay of chemical bonding and partial crystallographic disorder in the title compound.

## 2 Experimental Section

Samples with nominal compositions Mg_9_Pt and Mg_12_Pt were prepared from elemental Mg (granules; Alfa Aesar, 99.98%) and Pt (powder; Alfa Aesar, 99.9%). Pt powder was added on the already induction-melted Mg granules on the bottom of a tantalum tube. The tantalum tube was welded and heated up to 1080 °C in a high-frequency furnace for about 5 min. The melted product was ground and compacted into a pellet, and the latter was placed in an alumina crucible and sealed into a tantalum tube for the annealing procedure. The sample after being cooled from 650 to 530 °C in 240 h was held at this temperature for 5 days. The reaction product consisted of a polycrystalline compacted gray pellet with a metallic appearance. The complete sample preparation was performed in an argon (Ar)-filled glovebox (MBraun, *p*(H_2_O/O_2_) < 0.1 ppm).

The study of the thermal behavior of Mg_29–*x*_Pt_4+*y*_ was carried out on a Netzsch DSC 404C Pegasus differential scanning calorimeter in a flowing Ar atmosphere (99.999%, 100 mL/min) with subsequent drying and oxygen post-purification via a Big Oxygen Trap (Trigon Technologies) and using a thermocouple type S. The analyzed sample mass was 30 mg and the rate of heating and cooling was 10.0 K/min. A closed Al_2_O_3_ crucible inside a sealed Ta ampoule was used during the measurements.

The chemical composition of the Mg_29–*x*_Pt_4+*y*_ phase was determined with wavelength-dispersive X-ray spectroscopy (WDXS), which was performed on the electron microprobe Cameca SX100 using Mg_2_Sn and elemental Pt as standards for the intensity measurements of the Mg Kα and Pt Lα X-ray lines at an acceleration voltage of 20 kV. Applying the ZAF matrix correction model led to analytical totals of 100.6(2) wt %.

Powder XRD was performed with a Guinier-Huber Image Plate Camera G670, Cu Kα_1_ radiation (λ = 1.54056 Å) with LaB_6_ as internal standard. Single crystals of the phase were selected from the crushed annealed samples. The splitters were glued to thin glass fibers and were analyzed at room temperature using a Rigaku AFC7 diffraction system equipped with a Saturn 724+ CCD detector (Mo Kα radiation, λ = 0.71073 Å). The lattice parameters from the refinement of the powder and single-crystal data are in good agreement. Absorption correction was performed by a multi-scan procedure. All crystallographic calculations were performed with the program package WinCSD.^31^

The Dresden Grand ARM, a double-corrected JEM-ARM300F microscope, was used for aberration-corrected TEM and HAADF-STEM imaging at 300 kV.^32^ The images were recorded on a 4k × 4k pixel CCD array (Gatan US4000).

The all-electron, full-potential local-orbital method (FPLO) within the local density approximation^33^ and the Perdew–Wang parametrization^34^ was employed for quantum chemical calculations on four ordered models described in the main text. Experimentally obtained lattice parameters and atomic coordinates were employed for the calculations. Due to the large size of the unit cell with ca. 400 atoms, the calculations were extremely expensive in computation time.

The analysis of the chemical bonding in position space^35,36^ was performed by means of the electron localizability approach. For this purpose, the electron localizability indicator (ELI) in its ELI-D representation^37,38^ and the electron density (ED) were calculated with a specialized module in the FPLO code.^39^ The topologies of ELI-D and ED were evaluated by means of the program DGrid.^40^ The atomic charges from ED and bond populations for bonding basins from ELI-D were obtained by the integration of ED within the basins (space regions) bound by zero-flux surfaces in the corresponding gradient field. This procedure follows the quantum theory of atoms in molecules (QTAIM).^41^

## 3 Results and Discussion

The X-ray powder diffraction pattern of Mg_29–*x*_Pt_4+*y*_ synthesized from the elements (nominal sample composition, Mg_12_Pt) was indexed on the basis of a face-centered cubic unit cell with *a* = 20.1068(2) Å (Figure S1). The powder XRD pattern of the Mg-rich sample Mg_12_Pt showed small amounts of elemental Mg as impurity; this was also observed in the metallographic analysis (Figure S2). The powder XRD pattern of the Mg-deficient sample Mg_9_Pt showed reflections of the impurity Mg_3_Pt. The slightly smaller lattice parameter *a* = 20.1045(2) Å of the majority phase Mg_29–*x*_Pt_4+*y*_ indicates a possibly quite narrow homogeneity range of the latter. The compound is formed by the peritectic reaction at 576(2) °C from Mg_3_Pt and Mg-rich melt (Figures S2 and S3).

The crystal structure was solved using 2487 symmetry independent reflections from single-crystal diffraction data in the non-centrosymmetric space group *F*4̅3*m* (Table 1). The positions of three symmetry-independent Pt and nine Mg atoms (Mg1–Mg7, Mg9, Mg10, cf. Table 2) were easily refined, revealing a high level of ordering in the structure. The large atomic displacement parameter for the Mg8 site indicated its partial occupancy. Indeed, the refinement yields the site occupation factor SOF(Mg8) = 0.74(3). Consequently, the next closest Mg position has to be split into Mg11 (when Mg8 is present) and Mg12 (Mg8 is not occupied). The independent refinement of their occupancy factors reveals that at Mg11 there is more ED and at Mg12 there is less ED than required by the expected occupancy ratio of 0.74:0.26. In the case of Mg11, this leads to a rather unusual partial occupancy by Pt. The final refinement resulted in occupancies of 58% Mg + 5% Pt for Mg11 and 15% Mg for Mg12. The refinement converged to a final *R*_F_ value of 0.0262 (Table 1). A partial occupancy of the Mg8 and Mg12 sites by Mg and a mixed occupancy by Mg/Pt of the Mg11 site yielded refined compositions of 87.5 at. % Mg and 12.5 at. % Pt, in good agreement with the WDXS analysis, which shows 87.6(1) at. % Mg and 12.4(1) at. % Pt (Table S1 and Figure S2). The refined atomic coordinates together with the equivalent displacement parameters are listed in Table 2. The anisotropic atomic displacement parameters and the interatomic distances can be found in Tables S2 and S3, respectively. Another possibility to refine the structure is to consider the presence of a Pt atom in the 4*a* position (Figure S8, Tables S4–S6). As may be expected, in this case, the residual value *R*_F_ does not change markedly; it even increases slightly to 0.027, and the refined composition turns out to not change essentially (87.7 at. % Mg and 12.3 at. % Pt).

**Table 1:** Crystallographic Data for Mg_29–*x*_Pt_4+*y*_ (*x* = 0.47, *y* = 0.07)

composition	Mg_28.53_Pt_4.07_
space group	*F*4̅3*m*
Pearson symbol	*cF*391.2
formula units per unit cell, *Z*	12
unit cell parameter, *a*	20.1068(2) Å^a^
unit cell volume, *V*	8128.8(3) Å^3^
calculated density, ρ	3.63 g cm^–3^
crystal shape	irregular
crystal size	0.080 × 0.090 × 0.095 mm^3^
diffraction system	RIGAKU AFC7
detector	Saturn 724+ CCD
radiation, wavelength, λ	Mo Kα, 0.71073 Å
scan type; step per degree; *N*(images)	φ, 0.5, 1140
2θ_max_	79.96°
measured range in *hkl*	–35 ≤ *h* ≤ 36
	–36 ≤ *k* ≤ 24
	–22 ≤ *l* ≤ 36
absorption correction	multi-scan
absorption coefficient	22.61 mm^–1^
*T*(max)/*T*(min)	0.22/0.14
*N*(*hkl*) measured	41,127
*N*(*hkl*) unique	2487
*R*(int)	0.0507
*N*(*hkl*) observed	2446
observation criteria	*F(hkl*) ≥ 4σ[*F*(*hkl*)]
number of refined parameters	65
*R*_F_, *R*_w_	0.0262, 0.0273
largest diff. peak and hole (e^–^ Å^–3^)	–0.11/0.13

a Lattice parameter is refined from 106 reflections of the powder XRD pattern, Guinier technique, Cu Kα_1_ radiation, λ = 1.54056 Å.

**Table 2:** Atomic Coordinates and Isotropic Displacement Parameters for Mg_29–*x*_Pt_4+*y*_ (*x* = 0.47 and *y* = 0.07; Space Group *F*4̅3*m*)

atom	site	*x*/*a*	*y*/*b*	*z*/*c*	*U*_eq/iso_ (Å^2^)^a^
Pt1	16*e*	0.58148(1)	*x*	*x*	0.01292(4)
Pt2	16*e*	0.84958(1)	*x*	*x*	0.01218(4)
Pt3	16*e*	0.34382(1)	*x*	*x*	0.01207(4)
Mg1	16*e*	0.4480(2)	*x*	*x*	0.0156(4)
Mg2	24*g*	0.6438(2)	^1^/_4_	^1^/_4_	0.0139(6)
Mg3	24*f*	0.3171(2)	0	0	0.0195(8)
Mg4	48*h*	0.0993(1)	*x*	0.2283(2)	0.0214(5)
Mg5	48*h*	0.05124(9)	*x*	0.8398(2)	0.0182(5)
Mg6	24*g*	0.1327(2)	^1^/_4_	^1^/_4_	0.0175(7)
Mg7	48*h*	0.1939(1)	*x*	0.9814(2)	0.0166(5)
Mg8^b^	4*a*	0	0	0	0.019(3)
Mg9	48*h*	0.15489(9)	*x*	0.4771(1)	0.0153(4)
Mg10	48*h*	0.10690(8)	*x*	0.7154(1)	0.0145(4)
Mg11^b^	16*e*	0.0834(1)	*x*	*x*	0.0160(4)
Mg12^b^	16*e*	0.063(1)	*x*	*x*	0.018(5)

a *U*_eq_ = 4/3[*U*_11_(a*)^2^*a*^2^ + ... 2*U*_23_(b*)(c*)*bc* cos(α)]; *U*_iso_ for Mg8 and Mg12.

b The refined site occupancy factors (SOF): SOF(Mg8) = 0.74(3) Mg; SOF (Mg11) = 0.58(6) Mg + 0.050(7) Pt; SOF(Mg12) = 0.15(2) Mg.

The observed SOFs are understood as superposition of four different local ordering variants around the origin (all other positions are the same in all four models):

Model 1: Mg8 and Mg11 occupied by Mg (unit cell composition, Mg_348_Pt_48_);

Model 2: Mg8 non-occupied, Mg12 occupied by Mg (Mg_344_Pt_48_);

Model 3: Mg8 occupied by Mg, Mg11—by Pt (Mg_332_Pt_64_);

Model 4: Mg8 occupied by Pt, Mg11—by Mg (Mg_344_Pt_52_).

From the effective charges (*cf*. bonding analysis below), other models with simultaneous occupation of both sites by Pt or Pt at Mg11 and non-occupied Mg8 position are considered as less probable because of the local agglomeration of negative charged species. The results of bonding analysis are visualized using models all four models (QTAIM charges) or model 2 (ELI-D analysis).

**Figure 1:**
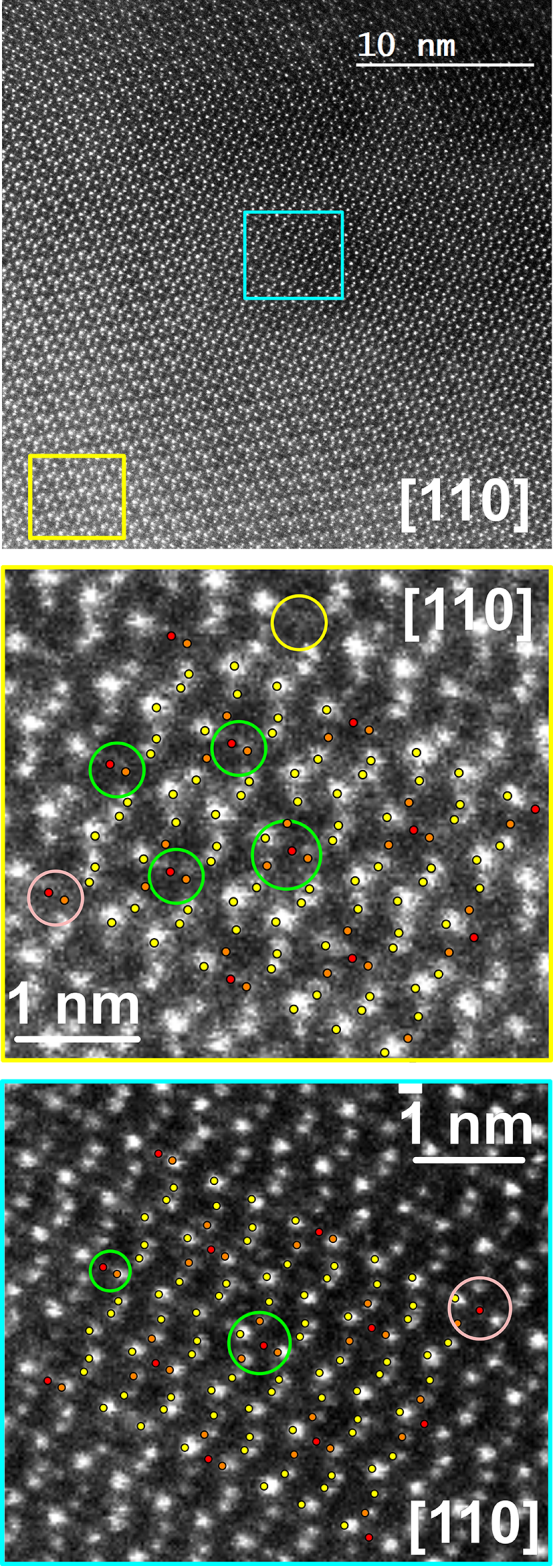
(Top panel) Atomic-resolution HAADF-STEM image of Mg_29–*x*_Pt_4+*y*_ along the [110] direction and selected regions—beam-untreated (yellow square, middle panel) and beam-treated (light-blue square, bottom panel)—with positions of the Pt atoms yellow—Pt1, Pt2, or Pt3, orange—Pt at the Mg11 position, red—Pt at the Mg8 position. The characteristic regions with predominant Pt ordering according to the models are tagged (cf. text): yellow circles—model 1, green circles—model 3, and light pink circles—model 4.

The appearance of local atomic arrangements corresponding to the four models mentioned above was verified by atomic-resolution TEM. The experimental HAADF-STEM image shows predominantly the positions of Pt atoms (atomic columns, Figure 1, top). The regions which were not exposed for a long time to the electron beam (Figure 1, middle) revealed besides the “regular” Pt atoms Pt1, Pt2, and Pt3, additional Pt at the positions Mg11 (model 3, more often, green circles) and Mg8 (model 4, quite rare, light pink circles), as well as regions where Pt was absent at these positions (models 1 and 2, yellow circles).

**Figure 2:**
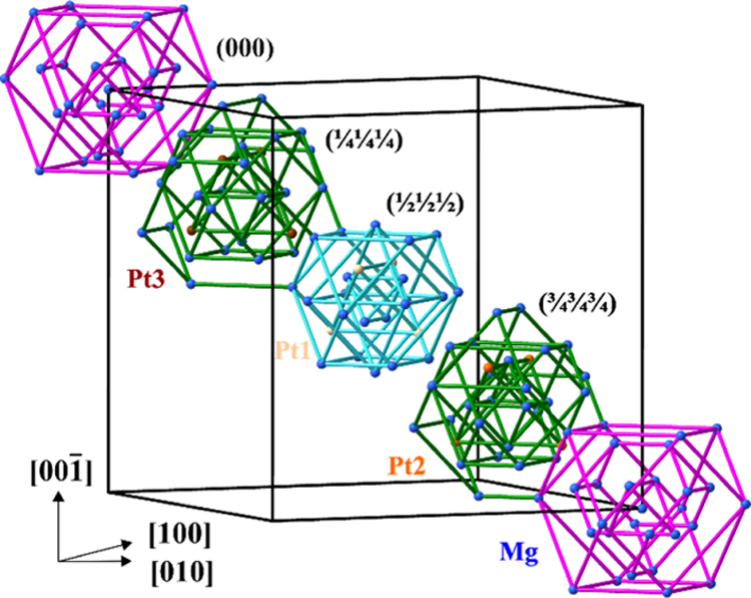
Arrangement of nested polyhedral units at high symmetry points in the unit cell of Mg_29–*x*_Pt_4+*y*_: α-Mn unit at (000), γ-brass unit at (^1^/_2_^1^/_2_^1^/_2_), and Ti_2_Ni units at (^1^/_4_^1^/_4_^1^/_4_) and (^3^/_4_^3^/_4_^3^/_4_). The first one contains only Mg atoms (model 1, cf. text), in the remaining units, the inner tetrahedron is formed by Pt1, Pt2, or Pt3 atoms (marked).

The crystal structure of Mg_29–*x*_Pt_4+*y*_ is close to being isopointal to the well-known Samson’s Mg_6_Pd (Mg_85.01_Pd_13.86_, or Mg_28.35_Pd_4.65_).^24^ In the prototype palladium (Pd) compound, the Mg8 position shows defects, and the Mg11 position is commonly occupied by Mg and Pd; only the split position Mg12 was not found in the original publication. According to the group–subgroup relations, Mg_29–*x*_Pt_4+*y*_ is a face-centered 2 × 2 × 2 superstructure of primitive Pd_8_Cd_43_ (space group *P*4̅3*m*, *a* = 9.9415 Å,^28^Figure S4), which—in turn—can be derived from the γ-brass arrangement. Usually the γ-brass derivatives are described by means of the nested polyhedral approach,^12−14^ analyzing the nearest environment of the high-symmetry points. In the case of Mg_29–*x*_Pt_4+*y*_, these are the point symmetry complexes 4̅3*m* at (000), (^1^/_4_^1^/_4_^1^/_4_), (^1^/_2_^1^/_2_^1^/_2_), and (^3^/_4_^3^/_4_^3^/_4_), as well as at translationally equivalent points (Figure 2). The first nested polyhedral unit centered at (000) is the so-called α-Mn-type unit.^12^ In this arrangement, a central atom (*C*) is surrounded by a tetrahedron (*T*), the second shell is formed by a truncated tetrahedron of 12 atoms (TT), and, finally, a cuboctahedron (CO) of 12 atoms forms the outer shell for a total of 29 atoms in the unit. The Ti_2_Ni-type nested polyhedral unit^11^ is centered at (^1^/_4_^1^/_4_^1^/_4_) and (^3^/_4_^3^/_4_^3^/_4_) and contains 34 atoms. The first shell is here an octahedron (OH), followed by an outer tetrahedron (OT) which is capping four of the eight triangular faces of the OH, the latter is inserted in a cuboctahedron CO (cantellated tetrahedron), and the final outermost shell is formed by a TT. Finally, a γ-brass nested polyhedral unit^11,42^ is found at (^1^/_2_^1^/_2_^1^/_2_). Here, the first shell is an empty regular inner tetrahedron, followed by an OT, the latter is inserted in a regular octahedron (OH), and the final shell is a cuboctahedron (CO), which completes the γ-brass unit.

**Figure 3:**
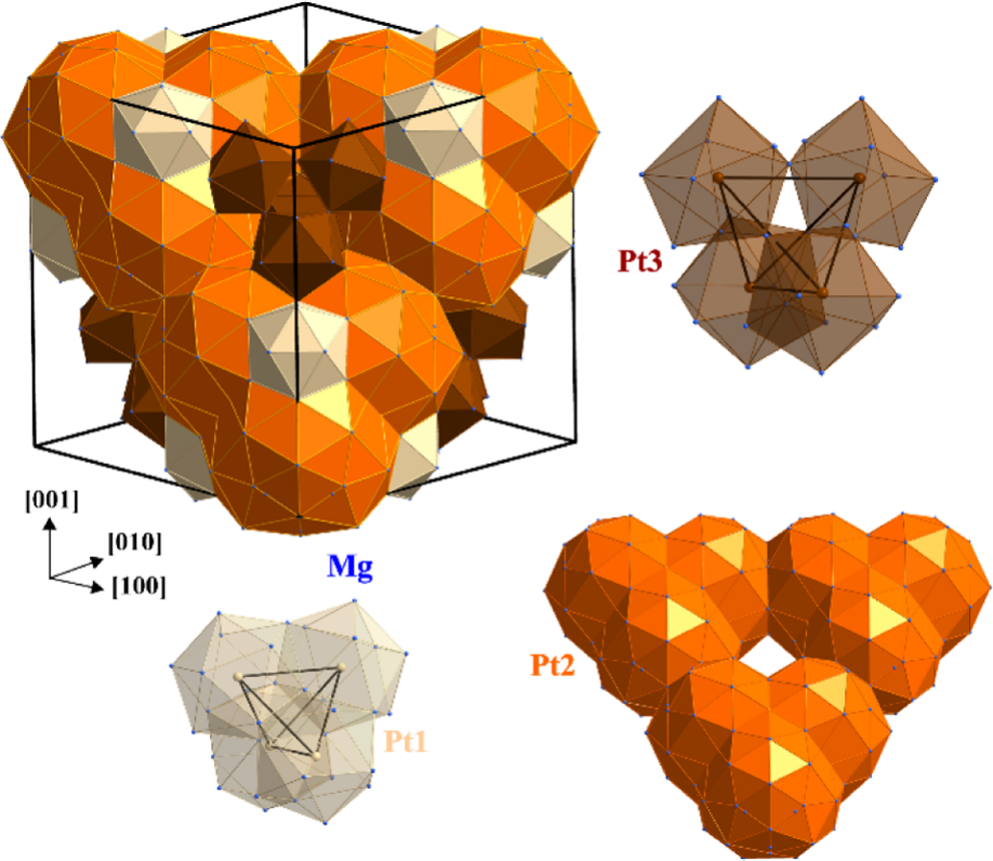
(Top left) Crystal structure of Mg_29–*x*_Pt_4+*y*_ as a packing of crystallographic clusters: (bottom left) Pearce clusters around Pt1 atoms; (top right) tetrahedral units of four PtMg_9_ tricapped trigonal prisms around Pt3; (bottom right) framework of Mackay clusters around OT(Pt2). Crystallographic clusters around Pt1, Pt2, and Pt3 atoms are drawn in beige, orange, and brown, respectively.

Starting with the nested polyhedral units at (^1^/_4_^1^/_4_^1^/_4_) and (^3^/_4_^3^/_4_^3^/_4_) and adding the following shells, the crystal structure of Mg_29–*x*_Pt_4+*y*_ can be interpreted as a framework of interpenetrating or face-sharing crystallographic Mackay clusters with Pt2 atoms forming the OT (Figures 3, S5, S9).^43^ The voids left in the unit cell are filled by tetrahedral units of four tricapped trigonal prisms PtMg_9_ centered by Pt3 atoms and by Pearce clusters^44^ centered by OT(Pt1). The latter cluster can be described as being built of four fused icosahedra having centers at the vertices of the OT tetrahedron (Figure 3). The crystallographic cluster approach opens an easier way to visualize the complex crystal structure of Mg_29–*x*_Pt_4+*y*_. Such crystallographic constructions allow the visualization of crystal structures, but leave open the question about their chemical roots. Thus, for the understanding of the reasons of the observed structural complexity and its influence on the properties of this material, further studies of chemical bonding are shown to be helpful.

To start with, the band structure calculations for the four structural models indicated that the observed disorder does not crucially influence the electronic density of states (DOS) for Mg_29–*x*_Pt_4+*y*_ (Figure S6). Being mainly defined by the Pt-d states, it shows one large region with a wide maximum between −4 and −3 eV (the regular Pt positions occupied only models 1 and 2) or between −4 and −2 eV (Pt at Mg8 or Mg11 sites, models 3 and 4). Less dependent on model and composition, a shallow minimum with non-negligible DOS around the Fermi level is found to indicate metal-like behavior for all models. Similar DOS features were recently observed in Be_21_Pt_5_^20^ and Be_5_Pt^21^ and interpreted as a result of complete filling of the valence electron band in a multi-center bond system combined with a strong charge transfer (cf. below).

Furthermore, Mg_29–*x*_Pt_4+*y*_ shows an average atomic volume of 20.8 Å^3^ that is significantly smaller than the atomic volume of 22.2 Å^3^ calculated for the experimental composition using the atomic volume of 23.25 Å^3^ for Mg and of 15.15 Å^3^ for Pt. Such a volume contraction indicates strong Mg–Pt interactions in the compound. In order to shed more light on the interplay between the described crystallographic features and the atomic interactions, further analysis of chemical bonding was performed employing quantum chemical techniques in the position space. Such an approach recently evolved to a powerful bonding-investigation tool, in particular for intermetallic compounds with a low electron number in the last shell per atom (<2) and multi-atomic bonding (e.g., refs (20)(21), and (30) and references therein).

**Figure 4:**
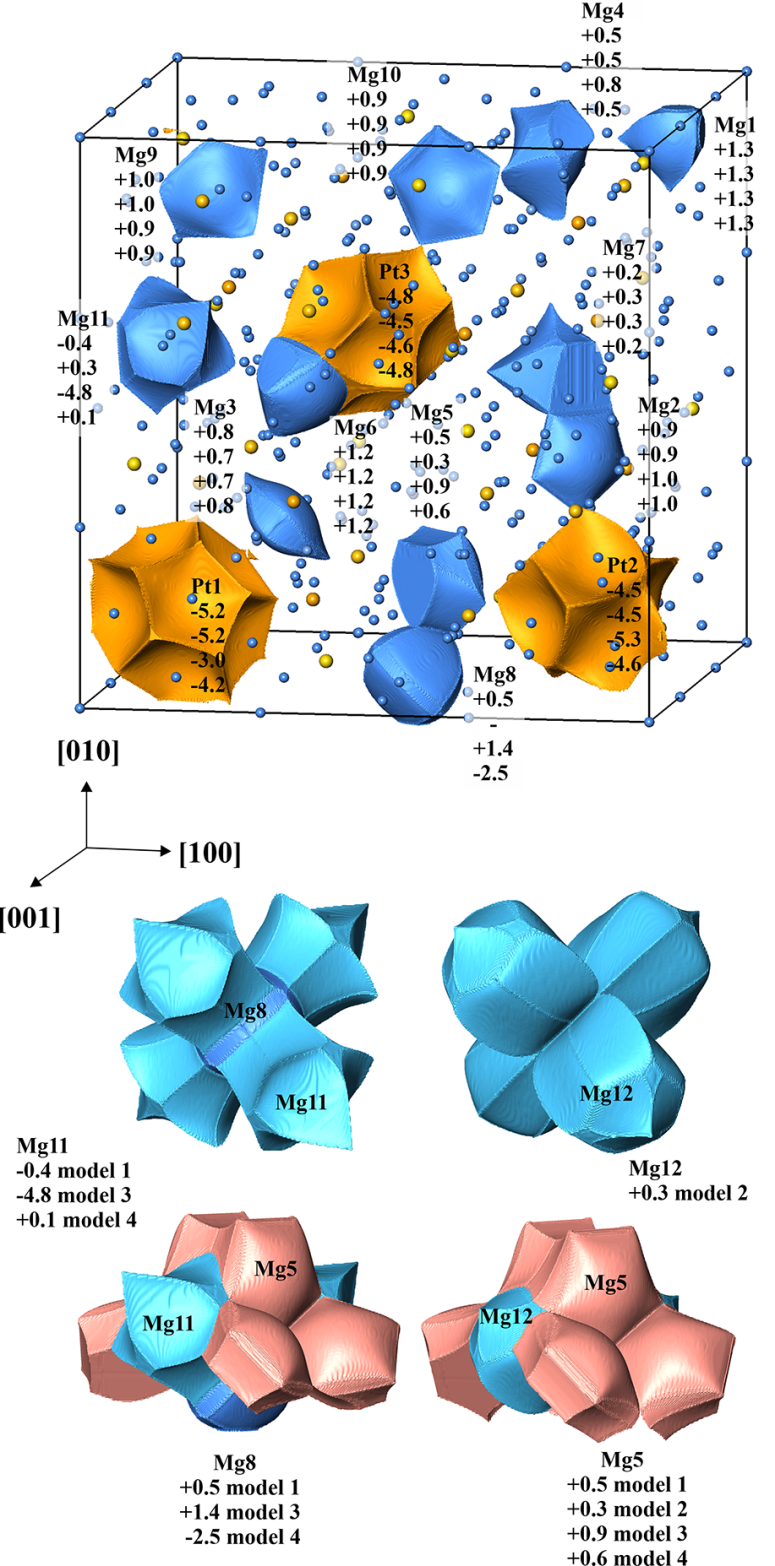
Charge transfer in Mg_29–*x*_Pt_4+*y*_ according to the QTAIM. (Top panel) Shapes and effective charges of the QTAIM atoms; for each atom, the first line gives the effective charge for model 1 with fully occupied Mg8 and Mg11 positions; the second line—for model 2; the third line—for model 3, and the fourth line—for model 4. (Middle and bottom panels) Arrangement of the atomic shapes around the origin (000) for the different models. Only the upper part of the arrangements is shown in the bottom panel for clarity.

Effective charges were evaluated within the QTAIM.^41^ For this purpose, the atomic shapes were obtained by employing the zero-flux surfaces in the gradient vector field of the ED, which form the boundaries of ED basins (QTAIM atoms, Figure 4). The so-obtained shapes are similar to those described for the chemically homologous compounds Be_21_Pt_5_,^20^ Be_5_Pt,^21^ and Mg_3_GaIr.^30^ The Mg atoms reveal compact convex polyhedra, and Pt shows large units with concave faces. Integration of the ED within these atomic basins yields their electronic population; subtracting the atomic number from the latter results in the effective charge. The latter follow the difference in the electronegativity of the elements. While Mg atoms typically have charges between +0.7 and +1.3 (for comparison, +1.2 to +1.4 in Mg_3_GaIr), Pt has a strong negative charge with −4.5 to −5.2 (cf. −4.8 to −5.7 in Be_21_Pt_5_ and −4.0 in Be_5_Pt). Most interesting are the calculated charge values in the region around the origin. In the case of model 1 (Mg8 and Mg11 are occupied by Mg, first line for atomic charges in Figure 4, top), the charge of Mg8 is rather small with +0.5, the next neighboring Mg11 has an even negative charge, and its neighbor Mg7 also has a reduced positive charge of +0.2 only. All these indicate an unfavorable agglomeration of particles with positive charge in this region in the sense of second Pauling’s structural principle for ionic materials (local electroneutrality).^45^ Indeed, removing Mg8 (model 2, second line for atomic charges in Figure 4, top) already improves the situation: Mg12 (split of Mg11), Mg5, and Mg7 obtain all small positive charges. The appearance of Pt at the Mg11 site (model 3, third line for atomic charges in Figure 4, top) indeed improves the situation: both Mg8 and Mg5 show clearly positive charges of +1.4 and +0.9, respectively; Mg7 remains slightly positive (+0.3). A similar effect has the occupation of the Mg8 position by Pt (model 4, fourth line for atomic charges in Figure 4, top): Mg11, Mg5, and Mg7 positions show positive charges. The results of effective charge calculations mentioned above allow us to understand the appearance of Pt atoms at the Mg11 and Mg8 positions indicated by the crystal structure refinements and TEM study.

**Figure 5:**
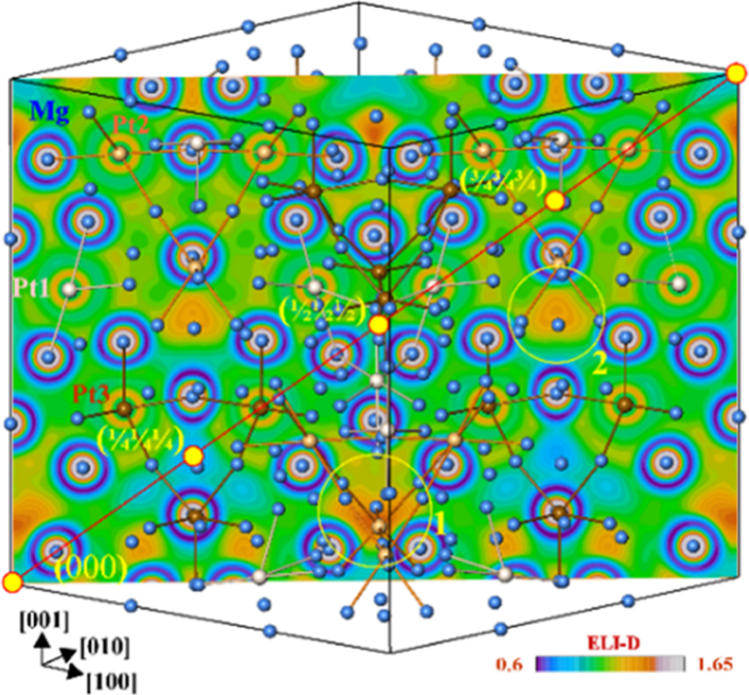
ELI-D distribution in the (1̅ 1 0) plane of the Mg_29–*x*_Pt_4+*y*_ unit cell (model 2). Positions of the four nested polyhedron clusters shown by filled yellow circles do not reveal ELI-D attractors. Two regions with the maximal values of ELI-D in this plane are shown with empty yellow circles. The shortest Pt3–Mg distances (brown) are shown for orientation.

**Figure 6:**
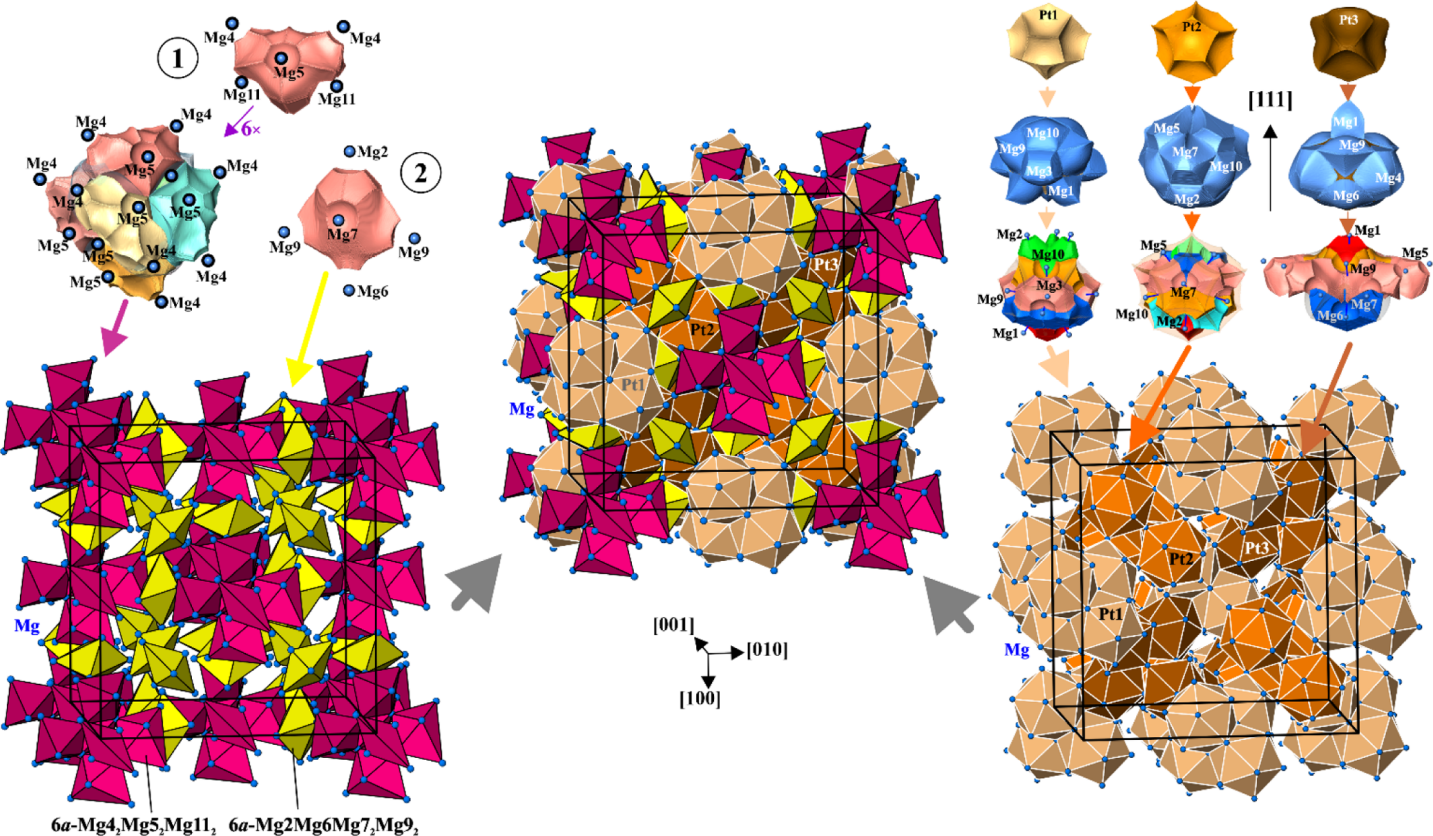
Organization of the chemical bonding in Mg_29–*x*_Pt_4+*y*_ (model 2). (Top left) Two types of homoatomic 6*a*-bonds (cf. regions 1 and 2 in Figure 5) involving only Mg atoms (light pink shapes); six items of the first kind form a large edge-bridged tetrahedron centered at (000); the second type of six atomic bonds yields a small arrangement formed by Mg only. (Bottom left) Six atomic bonds in the unit cell represented by isolated pink and yellow polyhedrons. (Top right) Atomic arrangements around the Pt atoms based on heteroatomic interactions: upper line—the Pt QTAIM shapes; middle line—the Pt atoms surrounded by the Mg ligands in the QTAIM representation; bottom line—the basins of multi-atomic bonds involving Pt atoms (filled shapes of different colors) located in most part within the QTAIM shapes of Pt atoms (transparent shapes). (Bottom right) Super-tetrahedrons formed by atomic arrangements around Pt atoms and centered at (^1^/_2_^1^/_2_^1^/_2_), (^1^/_4_^1^/_4_^1^/_4_), and (^3^/_4_^3^/_4_^3^/_4_) for Pt1, Pt2, and Pt3, respectively. (Middle panel) Interconnection of the Pt-based super-tetrahedrons by homoatomic 6*a*-bonds in the crystal structure of Mg_29–*x*_Pt_4+*y*_.

Recent analysis of chemical bonding, employing the electron localizability approach,^35−38^ in the chemically related compound Be_21_Pt_5_ with a crystal structure of similar complexity showed that the nested polyhedral units mark places of the multiatomic bonding, for example, the ELI-D reveals maxima at the centers of nested polyhedral units in Be_21_Pt_5_: half of the γ-brass units reflect 8- and 10-atomic bonds and the second half of 4- and 5-atomic bonds. In contrast, the ELI-D distribution in the (1̅10) plane in Mg_29–*x*_Pt_4+*y*_ (model 2, Figure 5) does not show maxima at the high-symmetry points with 43̅*m* point symmetry at (000), (^1^/_4_^1^/_4_^1^/_4_), (^1^/_2_^1^/_2_^1^/_2_), and (^3^/_4_^3^/_4_^3^/_4_). Evaluation of more than 1500 bonding attractors in the valence region in Mg_29–*x*_Pt_4+*y*_ (Figure S7) shows that the absolute ELI-D maxima in this plane are located in the vicinity of the origin [position (0 0 ≈0.1), region 1 in Figure 5], or close to the (^1^/_4_^1^/_4_ ≈^1^/_2_) point (region 2 in Figure 5). The first ELI-D attractor represents the six-atomic bond 6*a*-Mg4_2_Mg5_2_Mg11_2_ involving only Mg atoms (model 2). Six such bonds build an edge-bridged tetrahedron based on the OT(Mg11) around the origin of the unit cell (Figure 6, top left) leading to a large agglomeration of positively charged species in this region. The latter is partially reduced by the Pt replacement at Mg11 (model 3) and/or Mg8 (model 4) positions. The ELI-D attractor in region 2 represents another six-atomic Mg bond, 6*a*-Mg2Mg6Mg7_2_Mg9_2_, visualizing the second important building block in the title crystal structure. Both together form a system of isolated entities in the unit cell (Figure 6, bottom left).

Each of the Mg atoms (excluding Mg11) participating in the 6*a*-bonds also participates in the bonds involving Pt from the “regular” positions Pt1, Pt2, and Pt3. Indeed, on the one hand, the atomic basins of the Mg atoms fit well into the cavities of the atomic shapes of the corresponding Pt species, forming compact convex shapes (Figure 6, top right). On the other hand, most of the 3*a*-, 4*a*-, and 5*a*-ELI-D bonding basins involve “regular” Pt atoms as bond partners (Figure S7). These basins are, in most part, located within the atomic basins of Pt. Pt also contributes the larger part of the bond basin population, that is, these multi-atomic bonds are strongly polar (virtually ionic). This way, basic 13-atomic [Pt1Mg_12_] or [Pt2Mg_12_] and 11-atomic [Pt3Mg_10_] units are formed (Figure 6, top right) resembling the shortest interatomic distances (Table S3). The Pt-based units are condensed into a kind of super-tetrahedra by common faces (Pt1) or vertices (Pt2 and Pt3). The Pt2 and Pt3 units do not have common vertices, but both share vertices with the Pt1 unit (Figure 6, bottom right). The Pt1, Pt2, and Pt3 units are interconnected by the 6*a*-Mg2Mg6Mg7_2_Mg9_2_ bonds, and additionally Pt2 and Pt3 units are interlinked by the 6*a*-Mg4_2_Mg5_2_Mg7_2_ bonds (Figure 6, middle).

Such a local spatial separation of regions with different bonding types—homoatomic 6a-bonds and heteroatomic multicenter bonds with Pt participation—differs clearly from the bonding picture of the structurally and chemically similar compound Be_21_Pt_5_, where the multi- and hetero-atomic polar Be–Pt bonds are regularly distributed in the unit cell containing ca. 400 atoms. The most probable explanation is the larger electronegativity difference in the case of the title compound if compared with the Be–Pt phases. From this point of view, the bonding picture in Mg_29–*x*_Pt_4+*y*_ with spatially separated homo- and hetero-atomic interactions is rather new for the CMA compounds. One should note here that there are only few examples of the complete bonding analysis of the CMA compounds, which does not allow more general remarks. The expected appearance of further bonding patterns in the CMA family is a consequence of the systematic experimental research and discovery of its new representatives, for example, refs (46)–^48^.

## 4 Conclusions

The compound Mg_29–*x*_Pt_4+*y*_ belongs to the family of complex intermetallic compounds (CMA). Its cubic crystal structure with ca. 400 atoms in the unit cell can be geometrically described in terms of interpenetrating and face-sharing different crystallographic cluster types originating from nested polyhedral units located at the high-symmetry points in the unit cell, leaving open the chemical reasons for such complexity. Quantum chemical analysis of bonding in Mg_29–*x*_Pt_4+*y*_ employing the position space approach reveals a strong contribution of charge transfer and two types of multi-atomic bonds to the stabilization of the complex crystal structure. Structure building units around the Pt atoms (Pt1, Pt2, and Pt3) are formed by means of heteroatomic 3*a*-, 4*a*-, and 5*a*-bonds. The group of homoatomic 6*a*-bonds involving only Mg atoms is found to agglomerate around the origin of the unit cell and interconnect all three Pt-based units. Another group of 6*a*-bonds interlinks Pt2 and Pt3 units. Spatial separation of regions with different bonding features makes the key difference between Mg_29–*x*_Pt_4+*y*_ and other CMAs—derivatives of the γ-brass atomic arrangement—characterized by a homogeneous distribution of predominantly polar multi-center heteroatomic bonds in the unit cell.

## Supporting Information Available

Description of experimental and calculational techniques, electronic DOS, and details of structure evaluation and bonding analysis (PDF)

## References

[ref1] Sevov S. Zintl Phases. Intermetallic Compounds: Principles and Practice, Volume 3: Progress (Materials Science); Westbrook J. H., Fleischer R. L., Eds.; Wiley, 2002; p 1086ff.

[ref2] Ferro R.; Saccone A. Intermetallic Chemistry; Pergamon Materials Series; Elsevier, 2008.

[ref3] Grin Yu. Crystal Structure and Bonding in Intermetallic Compounds. Comprehensive Inorganic Chemistry II, 2nd ed.; Elsevier, 2013; pp 359–373.

[ref4] Steurer W.; Dshemuchadse J. Intermetallics: Structures, Properties, and Statistics; Oxford University Press, 2016; Vol. 26.

[ref5] Pauling L. Unsolved Problems of Structural Chemistry; Special Collections & Archives Research Center, OSU Libraries, Oregon State University, LP Speeches, Box 1947s.7 (Chem. Eng. News 1947, 25, 2970-2974), 1947.

[ref6] Feuerbacher M. Introduction to the science of Complex Metallic Alloys. In Complex Metallic Alloys. Fundamentals and Applications; Dubois J.-M., Bellin-Ferré E., Eds.; Wiley-VCH, 2010; pp 1–39.

[ref7] Urban K.; Feuerbacher M. Structurally complex alloy phases. J. Non-Cryst. Solids 2004, 334–335, 143–150. 10.1016/j.jnoncrysol.2003.11.029.

[ref8] Dshemuchadse J.; Jung D. Y.; Steurer W. Structural building principles of complex face-centered cubic intermetallics. Acta Crystallogr., Sect. B: Struct. Sci. 2011, 67, 269–292. 10.1107/s0108768111025390. 21775807

[ref9] Dshemuchadse J.; Steurer W. On the Symmetry and Composition of Complex Intermetallics. MRS Online Proc. Libr. 2012, 1517, 101 10.1557/opl.2012.1584.

[ref10] Weber T.; Dshemuchadse J.; Kobas M.; Conrad M.; Harbrecht B.; Steurer W. Large, larger, largest - a family of cluster-based tantalum copper aluminides with giant unit cells. I. Structure solution and refinement. Acta Crystallogr., Sect. B: Struct. Sci. 2009, 65, 308–317. 10.1107/s0108768109014001. 19461140

[ref11] Berger R. F.; Lee S.; Hoffmann R. A quantum mechanically guided view of Mg_44_Rh_7_. Chem.—Eur. J. 2007, 13, 7852–7863. 10.1002/chem.200700930. 17729218

[ref12] Chabot B.; Cenzual K.; Parthé E. Nested polyhedra units: a geometrical concept for describing complicated cubic structures. Acta Crystallogr., Sect. A: Found. Crystallogr. 1981, 37, 6–11. 10.1107/s0567739481000028.

[ref13] Hellner E.; Koch E. Cluster of framework considerations for the structures of Tl_7_Sb_2_, *α*-Mn, Cu_5_Zn_8_ and their variants Li_22_Si_51_, Cu_41_Sn_11_, Sm_11_Cd_45_, Mg_6_Pd and Na_6_Tl with octuple unit cells. Acta Crystallogr., Sect. A: Cryst. Phys., Diffr., Theor. Gen. Crystallogr. 1981, 37, 1–6. 10.1107/s0567739481000016.

[ref14] Bradley A. J.; Jones P. An X-ray investigation of the copper-aluminium alloys. J. Inst. Met. 1933, 51, 131–157.

[ref15] Henley C.; de Boissieu M.; Steurer W. Discussion on clusters, phasons and quasicrystal stabilisation. Philos. Mag. 2006, 86, 1131–1151. 10.1080/14786430500419395.

[ref16] Hume-Rothery W. Discussion on clusters, phasons and quasicrystal stabilisation. J. Inst. Met. 1926, 35, 307.

[ref17] Jones H. The theory of alloys in the *γ*-phase. Proc. R. Soc. London, Ser. A 1934, 144, 225–234. 10.1098/rspa.1934.0044.

[ref18] Mott N. F.; Jones H. The Theory of the Properties of Metals and Alloys; Clarendon Press: Oxford, 1936.

[ref19] Mizutani U. Hume-Rothery Rules for Structurally Complex Alloy Phases; CRC Press: Boca Raton, 2011.

[ref20] Amon A.; Ormeci A.; Bobnar M.; Akselrud L.; Avdeev M.; Gumeniuk R.; Burkhardt U.; Prots Yu.; Hennig Ch.; Leithe-Jasper A.; Grin Yu. Cluster formation in the superconducting complex intermetallic compound Be_21_Pt_5_. Acc. Chem. Res. 2018, 51, 214–222. 10.1021/acs.accounts.7b00561. 29313671

[ref21] Amon A.; Svanidze E.; Ormeci A.; König M.; Kasinathan D.; Takegami D.; Prots Yu.; Liao Y.-F.; Tsuei K.-D.; Tjeng L. H.; Leithe-Jasper A.; Grin Yu. Interplay of atomic interactions in the intermetallic semiconductor Be_5_Pt. Angew. Chem., Int. Ed. 2019, 58, 15928–15933. 10.1002/anie.201909782. PMC7754163 31483920

[ref22] Bonhomme F.; Yvon K. Cubic Mg_29_Ir_4_ crystallizing with an ordered variant of the Mg_6_Pd-type structure. J. Alloys Compd. 1995, 227, L1–L3. 10.1016/0925-8388(95)01673-2.

[ref23] Westin L.; Edshammar L.-E. Intermetallic compounds in the ruthenium-magnesium system. Chem. Scr. 1973, 3, 15–22.

[ref24] Sampson S. Complex cubic A_6_B compounds. II. The crystal structure of Mg_6_Pd. Acta Crystallogr., Sect. B: Struct. Sci. 1972, 28, 936–945. 10.1107/s0567740872003437.

[ref25] Makongo J.; Prots Yu.; Burkhardt U.; Niewa R.; Kudla C.; Kreiner G. A case study of complex metallic alloy phases: structure and disorder phenomena of Mg-Pd compounds. Philos. Mag. 2006, 86, 427–433. 10.1080/14786430500269212.

[ref26] Westin L.; Edshammar L.-E.; Krogh-Moe J.; Songstad J.; Pilotti Å. The Crystal Structure of Ir_7_Mg_44_. Acta Chem. Scand. 1972, 26, 3619–3626. 10.3891/acta.chem.scand.26-3619.

[ref27] Westin L.; Edshammar L.-E.; Leijonmarck M.; Mikhaiel S. A.; Engebretsen J. E.; Ehrenberg L. On the Crystal Structure of RhMg∼6. Acta Chem. Scand. 1971, 25, 1480–1481. 10.3891/acta.chem.scand.25-1480.

[ref28] Ferro R.; Rambaldi G. Research on the alloys of noble metals with the more electropositive elements. J. Less-Common Met. 1960, 2, 383–391. 10.1016/0022-5088(60)90047-3.

[ref29] Arnberg L. The structures of the γ-phases in the Pd-Cd and Pt-Cd systems. Acta Crystallogr., Sect. B: Struct. Sci. 1980, 36, 527–532. 10.1107/s0567740880003767.

[ref30] Sichevych O.; Prots Y.; Schnelle W.; Wagner F. R.; Grin Y. Polycation–Polyanion Architecture of the Intermetallic Compound Mg_3– x_Ga_1+ x_Ir. Molecules 2022, 27, 659 10.3390/molecules27030659. 35163924 PMC8840708

[ref31] Akselrud L.; Grin Yu. WinCSD: software package for crystallographic calculations (Version 4). J. Appl. Crystallogr. 2014, 47, 803–805. 10.1107/s1600576714001058.

[ref32] Ramlau R.; Grin Y.; Sawada H. Atomic resolution microscopy of intermetallic clathrates. JEOL News, Electron Opt. Instrum. 2016, 51, 2–6.

[ref33] Koepernik K.; Eschrig H. Full-potential nonorthogonal local-orbital minimum-basis band-structure scheme. Phys. Rev. B: Condens. Matter Mater. Phys. 1999, 59, 1743 10.1103/physrevb.59.1743.

[ref34] Perdew J. P.; Wang Y. Accurate and simple analytic representation of the electron-gas correlation energy. Phys. Rev. B: Condens. Matter Mater. Phys. 1992, 45, 13244 10.1103/physrevb.45.13244. 10001404

[ref35] Kohout M. A measure of electron localizability. Int. J. Quantum Chem. 2004, 97, 651–658. 10.1002/qua.10768.

[ref36] Wagner F. R.; Bezugly V.; Kohout M.; Grin Yu. Charge decomposition analysis of the electron localizability indicator: a bridge between the orbital and direct space representation of the chemical bond. Chem.—Eur. J. 2007, 13, 5724–5741. 10.1002/chem.200700013. 17458839

[ref37] Kohout M. Bonding indicators from electron pair density functionals. Faraday Discuss. 2007, 135, 43–54. 10.1039/b605951c. 17328423

[ref38] Kohout M.; Wagner F.; Grin Yu. Atomic shells from the electron localizability in momentum space. Int. J. Quantum Chem. 2006, 106, 1499–1507. 10.1002/qua.20925.

[ref39] Ormeci A.; Rosner H.; Wagner F. R.; Kohout M.; Grin Yu. Electron localization function in full-potential representation for crystalline materials. J. Phys. Chem. A 2006, 110, 1100–1105. 10.1021/jp054727r. 16420014

[ref40] Kohout M. DGrid, versions 4.6-5.0, 2018–2021.

[ref41] Bader R. F. W. Atoms in Molecules: A Quantum Theory; Oxford University Press: Oxford, 1999.

[ref42] Pankova A. A.; Blatov V. A.; Ilyushin G. D.; Proserpio D. M. γ-Brass Polyhedral Core in Intermetallics: The Nanocluster Model. Inorg. Chem. 2013, 52, 13094–13107. 10.1021/ic4019713. 24083847

[ref43] Kuo K. H. Mackay, anti-Mackay, double-Mackay, pseudo-Mackay, and related icosahedral shell clusters. Struct. Chem. 2002, 13, 221–230. 10.1023/A:1015847520094.

[ref44] Lord E.; Ranganathan S. Sphere packing, helices and the polytope {3, 3, 5 }. Eur. Phys. J. D 2001, 15, 335–343. 10.1007/s100530170149.

[ref45] Pauling L. The Nature of the Chemical Bond, 3rd ed.; Cornell University Press: Ithaka-New-York, 1986.

[ref46] Yox P.; Lebedev O. I.; Donadio D.; Kovnir K. Unprecedented superstructure in the type I family of clathrates. Chem. Commun. 2021, 57, 13780–13783. 10.1039/d1cc05167a. 34860234

[ref47] Vasquez G.; Latturner S. E. Metal flux growth of complex alkaline earth/rare earth metal silicides with a homologous series of metal phosphide structure types. Chem. Mater. 2018, 30, 6478–6485. 10.1021/acs.chemmater.8b02916.

[ref48] Chai P.; Abramchuk M.; Shatruk M. Synthesis, crystal structure, and magnetic properties of giant unit cell intermetallics R_117_Co_52+δ_Sn_112+γ_ (R = Y, La, Pr, Nd, Ho). Crystals 2016, 6, 165 10.3390/cryst6120165.

