# Supplementary material for: Mg29–xPt4+y: Chemical Bonding Inhomogeneity and Structural Complexity
Source: Inorg Chem. 2022 Sep 27;61(40):16148–55. doi: 10.1021/acs.inorgchem.2c02653 (PMC9554903; doi:10.1021/acs.inorgchem.2c02653)
Supplement: Supplementary file 1 — ic2c02653_si_001.pdf [file ic2c02653_si_001.pdf]

$Mg_{29-x}Pt_{4+y}$: Chemical Bonding Inhomogeneity and Structural Complexity

Laura Agnarelli, Yurii Prots, Reiner Ramlau, Marcus Schmidt, Ulrich Burkhardt, Andreas Leithe-Jasper, Yuri Grin*

*Prof. Yuri Grin
Max-Planck-Institut für Chemische Physik fester Stoffe
Nöthnitzer Str. 40
01187 Dresden
Germany

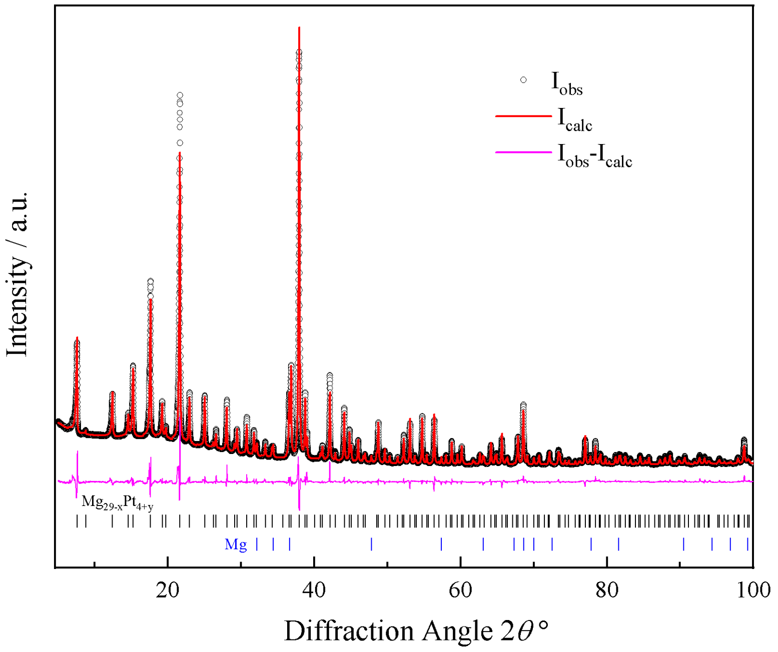

Figure 1S. XRPD pattern of the sample with nominal composition $Mg_{12}Pt$. annealed at 530 °C for 5 days (Cu Kα_1 radiation). Peak positions of the $Mg_{29-x}Pt_{4+y}$ phase and elemental Mg are marked with black and blue bars, respectively.

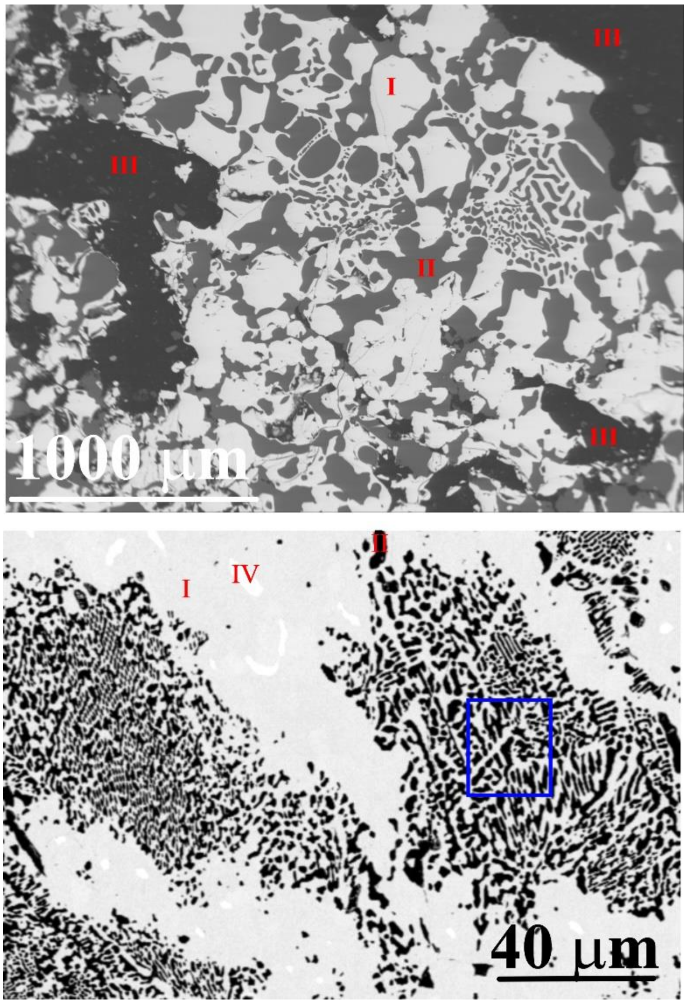

Figure 2S. (top) Microstructure (backscatter electron image) of the sample with nominal composition $Mg_{12}Pt$ annealed at 530 °C for 5 days (for powder X-ray diffraction pattern cf. Figure 1S). Bright regions represent the phase $Mg_{29-x}Pt_{4+y}$ (I), dark grey – the Mg-based solid solution (II), black - cavities filled up with resin (III). (bottom) Microstructure (backscatter electron image) of the as cast reaction product with starting composition Mg_9Pt containing the phase $Mg_{29-x}Pt_{4+y}$ (grey, I) with a little amount of Mg_3Pt (bright, IV) and Mg within the eutectic region (black, II); the bright grains of Mg_3Pt are surrounded by the extensive dark grey regions of $Mg_{29-x}Pt_{4+y}$, characteristic for a peritectic reaction; by further cooling during the synthesis, the eutectic $Mg_{29-x}Pt_{4+y}$ + Mg(Pt) with characteristic microstructure (blue rectangle) is formed.

Table 1S Chemical composition of the phase $Mg_{29-x}Pt_{4+y}$ determined by WDXS analysis on the electron microprobe (acceleration voltage of 20 kV). The measurement has been performed on the of the sample with nominal composition $Mg_{12}Pt$ annealed at 530°C for 5 days (cf. microstructure in Figure 2S).

	**Mass %**	**Atomic %**
Mg	47.1(1)	87.6(1)
Pt	53.5(1)	12.4(1)
Analytical total	100.6(2)	

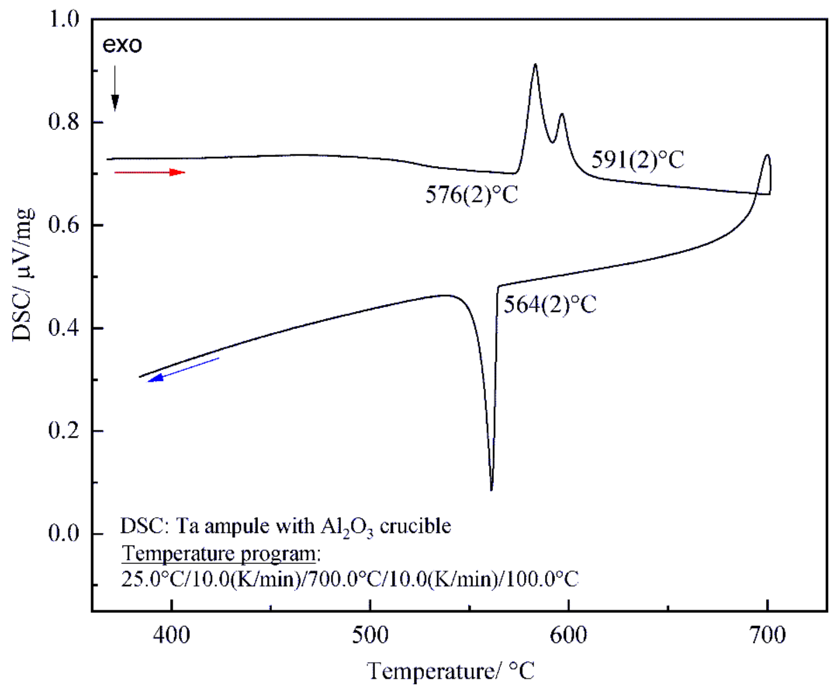

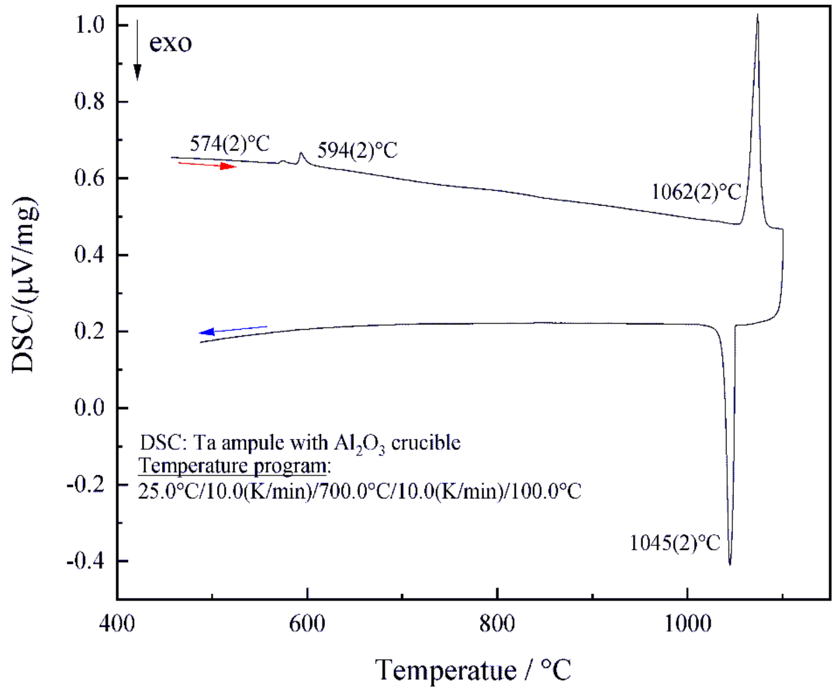

Figure 3S. Differential scanning calorimetry (DSC) of the samples with nominal composition $Mg_{92.31}Pt_{7.69}$ (top) and $Mg_{75.61}Pt_{24.39}$ (bottom) show that $Mg_{29-x}Pt_{4+y}$ forms peritectically from the neighbor phase Mg_3Pt and Mg-rich melt at 576(2) °C, while Mg_3Pt melts congruently at 1062(2) °C. This agrees with the metallographic analysis shown in Figure 2S. The onset temperatures are shown in the graph.

Table 2S. Anisotropic displacement parameters (Å^2) for $Mg_{29-x}Pt_{4+y}$ (x = 0.47, y = 0.07, space group $F\bar{4}3m$); for atom coordinates see Table 2.

**Atom**	U_{11}	U_{22}	U_{33}	U_{12}	U_{13}	U_{23}
Pt1	0.01294(6)	U_{11}	U_{11}	-0.00124(7)	U_{12}	U$_{12}$
Pt2	0.01220(6)	U_{11}	U_{11}	-0.00010(7)	U_{12}	U_{12}
Pt3	0.01208(6)	U_{11}	U_{11}	0.00081(7)	U_{12}	U_{12}
Mg1	0.0155(7)	U_{11}	U_{11}	0.0011(8)	U_{12}	U_{12}
Mg2	0.0130(13)	0.0144(8)	U_{22}	0	0	0.0009(10)
Mg3	0.024(2)	0.0176(10)	U_{22}	0	0	0.0013(13)
Mg4	0.0208(7)	U_{11}	0.0224(12)	0.0016(10)	-0.0017(7)	U_{13}
Mg5	0.0164(6)	U_{11}	0.0216(11)	-0.0010(8)	0.0003(6)	U_{13}
Mg6	0.025(2)	0.0139(9)	*U22*	0	0	0.0044(11)
Mg7	0.0171(6)	U_{11}	0.0159(10)	0.0005(8)	0.0006(6)	U_{13}
Mg9	0.0135(5)	U_{11}	0.0191(10)	-0.0004(9)	0.0009(6)	U_{13}
Mg10	0.0148(6)	U_{11}	0.0143(9)	-0.0014(7)	-0.0023(5)	U_{13}
Mg11	0.0163(7)	U_{11}	U_{11}	-0.0005(8)	U_{12}	U_{12}

Table 3S. Interatomic distances in the crystal structure of $Mg_{29-x}Pt_{4+y}$. Lattice parameter is listed in Table 1, for atom coordinates see Table 2.

Atoms		Distances (Å)	Atoms		Distances (Å)
Pt1	3 Mg10	2.787(3)	Mg6	2 Pt3	2.7093(8)
	3 Mg1	2.812(3)		4 Mg4	3.135(2)
	3 Mg9	2.960(2)		4 Mg6	3.335(4)
	3 Mg3	3.087(3)		2 Mg7	3.436(4)
Pt2	3 Mg5	2.827(2)		2 Mg9	3.491(3)
	3 Mg2	2.8349(3)	Mg7	1 Pt2	2.923(3)
	3 Mg7	2.923(3)		1 Mg2	2.980(4)
	3 Mg10	2.969(2)		2 Mg5	3.019(3)
Pt3	3 Mg9	2.680(3)		2 Mg10	3.088(3)
	3 Mg6	2.7093(8)		2 Mg4	3.117(3)
	3 Mg4	2.832(3)		1 Mg7	3.193(3)
	1 Mg1	3.629(3)		2 Mg9	3.250(3)
Mg1	3 Pt1	2.812(3)		1 Mg6	3.436(4)
	3 Mg1	2.956(4)		1 Mg11	3.752(3)
	3 Mg9	2.984(3)		1 Mg12	4.06(2)
	3 Mg3	3.019(5)	Mg8	4 Mg12	2.20(2)
	1 Pt3	3.629(3)		4 Mg11	2.901(3)
Mg2	2 Pt2	2.8349(3)		12 Mg5	3.535(3)
	2 Mg7	2.980(4)	Mg9	1 Pt3	2.680(3)
	4 Mg2	3.020(4)		1 Pt1	2.960(2)
	4 Mg10	3.052(2)		1 Mg1	2.984(3)
Mg3	2 Mg1	3.019(5)		2 Mg4	3.021(3)
	2 Pt1	3.087(3)		2 Mg10	3.037(3)
	2 Mg10	3.109(2)		2 Mg3	3.198(2)
	4 Mg9	3.198(2)		2 Mg7	3.250(3)
	2 Mg4	3.340(3)		1 Mg6	3.491(3)
	2 Mg5	3.475(5)	Mg10	1 Pt1	2.787(3)
Mg4	1 Pt3	2.832(3)		1 Mg5	2.961(4)
	2 Mg11	2.949(4)		1 Pt2	2.969(2)
	2 Mg9	3.021(3)		2 Mg9	3.037(3)
	2 Mg7	3.117(3)		2 Mg2	3.052(2)
	2 Mg 6	3.135(2)		2 Mg10	3.084(3)
	1 Mg3	3.340(3)		2 Mg7	3.088(3)
	2 Mg5	3.459(3)		1 Mg3	3.109(2)
	1 Mg12	3.47(2)	Mg11	1 Mg12	0.70(2)
	2 Mg4	3.669(4)		1 Mg8	2.901(3)
Mg5	1 Pt2	2.827(2)		3 Mg4	2.949(4)
	1 Mg5	2.914(3)		6 Mg5	3.182(3)
	1 Mg10	2.961(4)		3 Mg7	3.752(3)
	2 Mg7	3.019(3)	Mg12	1 Mg11	0.70(2)
	2 Mg12	3.03(2)		1 Mg8	2.20(2)
	2 Mg5	3.098(4)		6 Mg 5	3.03(2)
	2 Mg11	3.182(3)		3 Mg4	3.47 (2)
	2 Mg4	3.459(3)		3 Mg12	3.59(3)
	1 Mg3	3.475(5)		3 Mg7	4.06(2)
	1Mg8	3.535(5)			

Table 4S. Atomic positions and displacement parameters of $Mg_{29-x}Pt_{4+y}$ (x = 0.34, y = 0.024, Pt at Mg8 site).

Atom	Site	*S.O.F.*	*x/a*	*y/b*	*z/c*	$U_{eq/iso}$ [a] (Å^2)
Pt1	16*e*	1	0.58148(1)	*x*	*x*	0.01295(4)
Pt2	16*e*	1	0.84958(1)	*x*	*x*	0.01220(4)
Pt3	16*e*	1	0.34382(1)	*x*	*x*	0.01208(4)
Mg1	16*e*	1	0.4479(2)	*x*	*x*	0.0154(4)
Mg2	24*g*	1	0.6438(2)	¼	¼	0.0138(6)
Mg3	24*f*	1	0.3170(3)	0	0	0.0197(8)
Mg4	48*h*	1	0.0992(1)	*x*	0.2282(2)	0.0214(5)
Mg5	48*h*	1	0.05125(10)	*x*	0.8397(2)	0.0182(5)
Mg6	24*g*	1	0.1327(2)	¼	¼	0.0175(7)
Mg7	48*h*	1	0.1938(1)	*x*	0.9814(2)	0.0167(5)
Mg8	4*a*	0.072(4) Pt	0	0	0	0.020(3)
Mg9	48*h*	1	0.15491(9)	*x*	0.4771(1)	0.0154(4)
Mg10	48*h*	1	0.10688(9)	*x*	0.7153(1)	0.0146(4)
Mg11	16e	0.88(2) Mg	0.0832(2)	*x*	*x*	0.0103(6)
Mg12	16*e*	0.12(2) Mg	0.063(1)	*x*	*x*	0.009(8)

[a]$U_{eq} = 4/3\ [U_{11}\ (a^*)^2\ a^2 + \ldots\ 2U_{23}\ (b^*)\ (c^*)\ b\ c\ \cos(\alpha)]$; U_{iso} for Mg8 and Mg12.

Table 5S. Anisotropic displacement parameters (Å^2) of $Mg_{29-x}Pt_{4+y}$ (*x* = 0.34, *y* = 0.024, Pt at Mg8 site, atom coordinates are listed in Table 4S).

**Atom**	U_{11}	U_{22}	U_{33}	U_{12}	U_{13}	U_{23}
Pt1	0.01294(6)	U_{11}	U_{11}	-0.00124(7)	U_{12}	U_{12}
Pt2	0.01220(6)	U_{11}	U_{11}	-0.00010(7)	U_{12}	U_{12}
Pt3	0.01208(6)	U_{11}	U_{11}	0.00081(7)	U_{12}	U_{12}
Mg1	0.0155(7)	U_{11}	U_{11}	0.0011(8)	U_{12}	U_{12}
Mg2	0.0130(13)	0.0144(8)	U_{22}	0	0	0.0009(10)
Mg3	0.024(2)	0.0176(10)	U_{22}	0	0	0.0013(13)
Mg4	0.0208(7)	U_{11}	0.0224(12)	0.0016(10)	-0.0017(7)	U_{13}
Mg5	0.0164(6)	U_{11}	0.0216(11)	-0.0010(8)	0.0003(6)	U_{13}
Mg6	0.025(2)	0.0139(9)	U_{22}	0	0	0.0044(11)
Mg7	0.0171(6)	U_{11}	0.0159(10)	0.0005(8)	0.0005(6)	U_{13}
Mg9	0.0135(6)	U_{11}	0.0191(10)	-0.0004(9)	0.0009(6)	U_{13}
Mg10	0.0148(6)	U_{11}	0.0143(10)	-0.0014(7)	-0.0023(5)	U_{13}
Mg11	0.0163(7)	U_{11}	U_{11}	-0.0005(8)	U_{12}	U_{12}

Table 6S. Interatomic distances in the crystal structure of $Mg_{29-x}Pt_{4+y}$ (Pt at Mg8 site, atom coordinates are listed in Table 4S).

**Atoms**		**Distances (Å)**	**Atoms**		**Distances (Å)**	**Atoms**		**Distances (Å)**
Pt1	3 Mg10	2.790(3)		1 Mg3	3.478(5)	Mg12	1 Mg8	2.14(2)
	3 Mg1	2.814(3)		1 Mg8	3.538(3)		6 Mg5	3.02(2)
	3 Mg9	2.963(2)	Mg6	2 Pt3	2.7116(8)		3 Mg12	3.50(3)
	3 Mg3	3.089(3)		4 Mg4	3.138(2)		3 Mg4	3.52(2)
Pt2	3 Mg5	2.829(2)		4 Mg6	3.338(4)		3 Mg7	4.10(2)
	3 Mg2	2.8374(3)		2 Mg7	3.439(5)		3 Mg11	4.14(2)
	3 Mg7	2.926(3)		2 Mg9	3.494(3)			
	3 Mg10	2.971(2)	Mg7	1 Pt2	2.926(3)			
Pt3	3Mg9	2.682(3)		1 Mg2	2.983(4)			
	3 Mg6	2.7116(8)		2 Mg5	3.021(3)			
	3 Mg4	2.834(3)		2 Mg10	3.091(3)			
	1 Mg1	3.631(3)		2 Mg4	3.120(3)			
Mg1	3 Pt1	2.814(3)		1 Mg7	3.196(3)			
	3 Mg1	2.960(4)		2 Mg9	3.253(3)			
	3 Mg9	2.986(3)		1 Mg6	3.439(4)			
	3 Mg3	3.022(5)		1 Mg11	3.575(4)			
	1 Pt3	3.631(3)		1 Mg12	4.10(2)			
Mg2	2 Pt2	2.8374(3)	Mg8	4 Mg12	2.14(2)			
	2 Mg7	2.983(4)		4 Mg11	2.900(3)			
	4 Mg2	3.022(4)		12 Mg5	3.538(3)			
	4 Mg10	3.055(2)	Mg9	1 Pt3	2.682(3)			
Mg3	2 Mg1	3.022(5)		1 Pt1	2.963(2)			
	2 Pt1	3.089(3)		1 Mg1	2.986(3)			
	2 Mg10	3.112(2)		2 Mg4	3.023(4)			
	4 Mg9	3.201(2)		2 Mg10	3.040(3)			
	2 Mg4	3.343(3)		2 Mg3	3.201(2)			
	2 Mg5	3.478(5)		2 Mg7	3.253(3)			
Mg4	1 Pt3	2.834(3)		1 Mg6	3.494(3)			
	1 Mg11	2.955(4)		2 Mg9	3.756(3)			
	2 Mg9	3.023(3)	Mg10	1 Pt1	2.790(3)			
	2 Mg7	3.120(3)		1Mg5	2.963(4)			
	2 Mg 6	3.138(2)		1 Pt2	2.971(2)			
	1 Mg3	3.343(3)		2 Mg9	3.040(3)			
	2 Mg5	3.462(3)		2 Mg2	3.055(2)			
	1 Mg12	3.52(2)		2 Mg10	3.087(3)			
	2 Mg4	3.672(4)		2 Mg7	3.091(3)			
Mg5	1 Pt2	2.829(2)		1 Mg3	3.112(2)			
	1 Mg5	2.917(3)	Mg11	1 Mg12	0.76(2)			
	1 Mg10	2.963(4)		1Mg8	2.900(3)			
	2 Mg7	3.021(3)		3 Mg4	2.955(4)			
	2 Mg12	3.02(2)		6 Mg5	3.183(4)			
	2 Mg5	3.101(4)		3 Mg7	3.757(4)			
	2 Mg11	3.183(4)		3 Mg12	4.14(2)			
	2 Mg4	3.462(3)	Mg12	1 Mg11	0.76(2)			

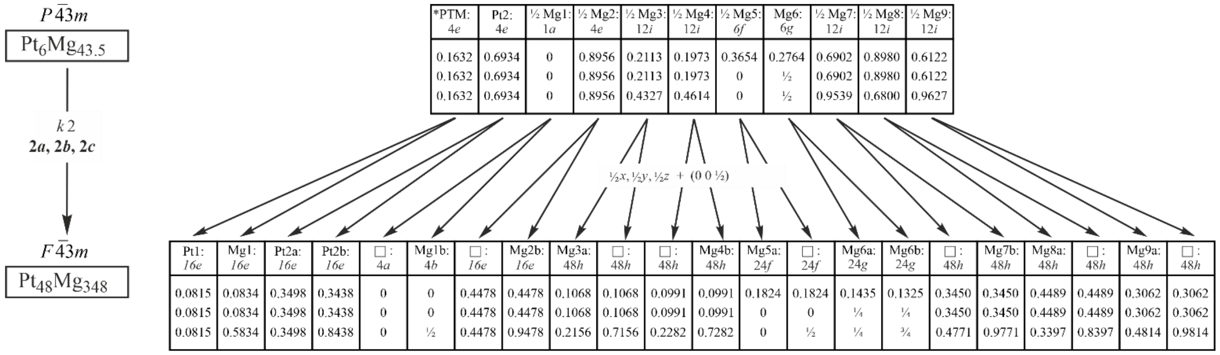

*PTM: 4e	Pt2: 4e	½ Mg1: 1a	½ Mg2: 4e	½ Mg3: 12i	½ Mg4: 12i	½ Mg5: 6f	Mg6: 6g	½ Mg7: 12i	½ Mg8: 12i	½ Mg9: 12i
0.1632	0.6934	0	0.8956	0.2113	0.1973	0.3654	0.2764	0.6902	0.8980	0.6122
0.1632	0.6934	0	0.8956	0.2113	0.1973	0	½	0.6902	0.8980	0.6122
0.1632	0.6934	0	0.8956	0.4327	0.4614	0	½	0.9539	0.6800	0.9627

Pt1: 16e	Mg1: 16e	Pt2a: 16e	Pt2b: 16e	□: 4a	Mg1b: 4b	□: 16e	Mg2b: 16e	Mg3a: 48h	□: 48h	□: 48h
0.0815	0.0834	0.3498	0.3438	0	0	0.4478	0.4478	0.1068	0.1068	0.0991
0.0815	0.0834	0.3498	0.3438	0	0	0.4478	0.4478	0.1068	0.1068	0.0991
0.0815	0.5834	0.3498	0.8438	0	½	0.4478	0.9478	0.2156	0.7156	0.2282

Mg4b: 48h	Mg5a: 24f	□: 24f	Mg6a: 24g	Mg6b: 24g	□: 48h	Mg7b: 48h	Mg8a: 48h	□: 48h	Mg9a: 48h	□: 48h
0.0991	0.1824	0.1824	0.1435	0.1325	0.3450	0.3450	0.4489	0.4489	0.3062	0.3062
0.0991	0	0	¼	¼	0.3450	0.3450	0.4489	0.4489	0.3062	0.3062
0.7282	0	½	¼	¾	0.4771	0.9771	0.3397	0.8397	0.4814	0.9814

*PTM: = ½ Pt + ½ Mg

Figure 4S. $Pd_{48}Cd_3$ crystallizes with a *P* centered cubic unit cell, with lattice parameter a=9.9415(5) Å which is approximately half of the lattice parameter of $Mg_{28.59}Pt_{4.06}$ (20.1068(2)) Å. Moreover, comparing the distribution of the atoms in the unit cell of the two compounds, some similarities can be observed. In addition to this, by the loss of centering translations, $F\bar{4}3m$ is a maximal *klassengleiche* non-isomorphic subgroup with enlarged unit cell of $P\bar{4}3m$. Starting from these assumptions, using the same single crystal dataset of $Mg_{28.59}Pt_{4.06}$, the latter was first of all transformed in a reduced cubic unit cell with a= 10.0623(1) Å and it was refined in the space group $P\bar{4}3m$. The obtained refined crystal structure (R_F= 0.0210 R_w= 0.0408) shows a composition $Pt_6Mg_{43.50}$ with 8 of the 11 positions half-occupied and one position showing a mixed occupancy of Mg and Pt. Performing a *klassengleiche* transformation of order 2 with the indicated coordination transformations in the following Bärnighausen tree, the crystal structure of $Mg_{29}Pt_4$ can be derived.

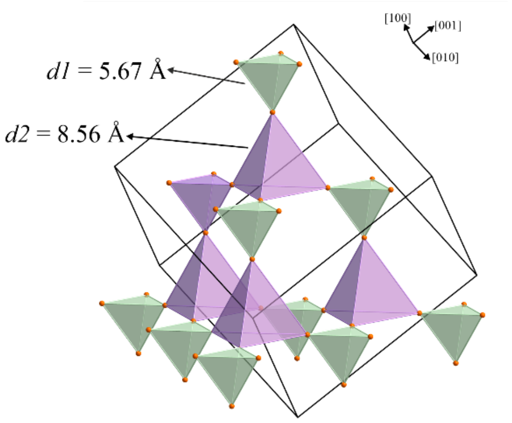

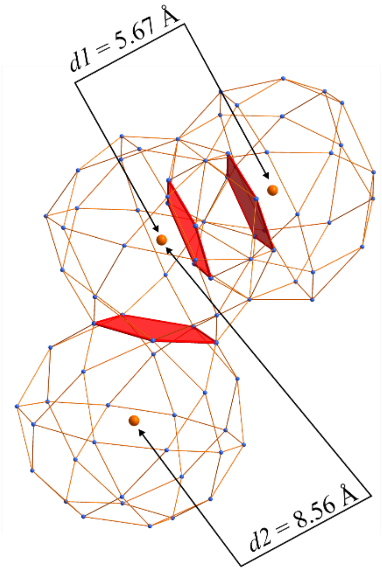

Figure 5S. In the crystal structure of $Mg_{29-x}Pt_{4+y}$ each Pt2 atom has three Pt2 neighbors at distance of 5.67 Å and three Pt2neighbors at longer distance of 8.56 Å, resulting in disposition of Pt2 atoms according to a diamond net of $(Pt2)_4$ tetrahedra (upper panel). Around Pt2 a Mackay cluster can be built and in particular the Pt2 atoms at distance 5.67 Å form Mackay clusters which are interpenetrating through the pentagonal faces of the icosidodecahedron, while the ones at distance equal to 8.56 Å are sharing the pentagonal faces (bottom panel).

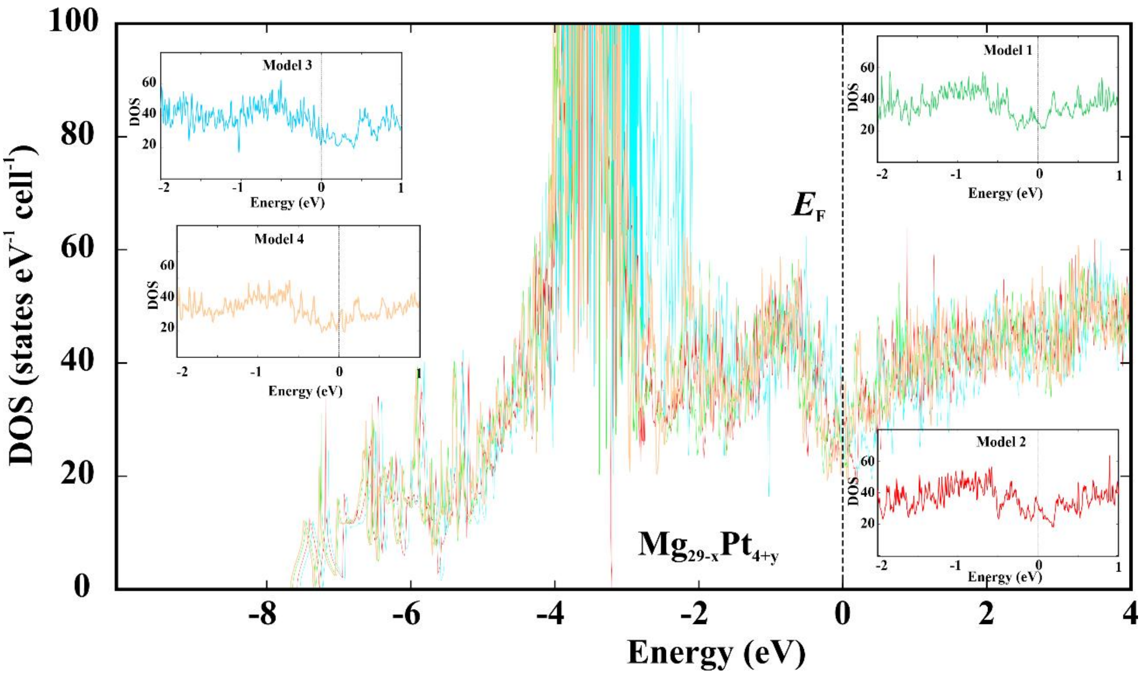

Figure 6S. Total electronic density of states (DOS) for $Mg_{29-x}Pt_{4+y}$: green - model 1, composition $Mg_{29}Pt_4$, red - model 2, composition $Mg_{28.75}Pt_4$; inserts show the regions around the Fermi level for model 1 (top right), model 2 (bottom right) and model 3 (top left).

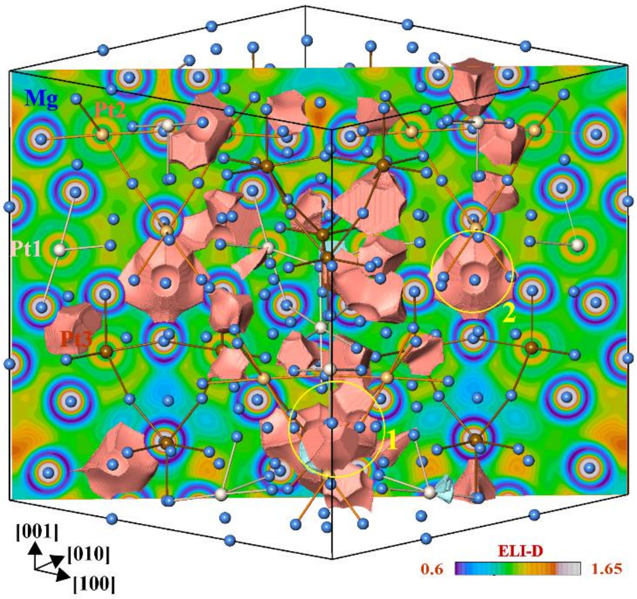

Figure 7S. ELI-D bonding basins in $Mn_{29-x}Pt_{4+y}$ (model 2): 1 and 2 mark the basins of the six-atomic Mg_6 bonds with the maxima of ELI-D highlighted in Figure 5. The remaining basins represent three-, four- or five-atomic interactions, most of them with participation of the platinum atoms, as presented in Figure 6.

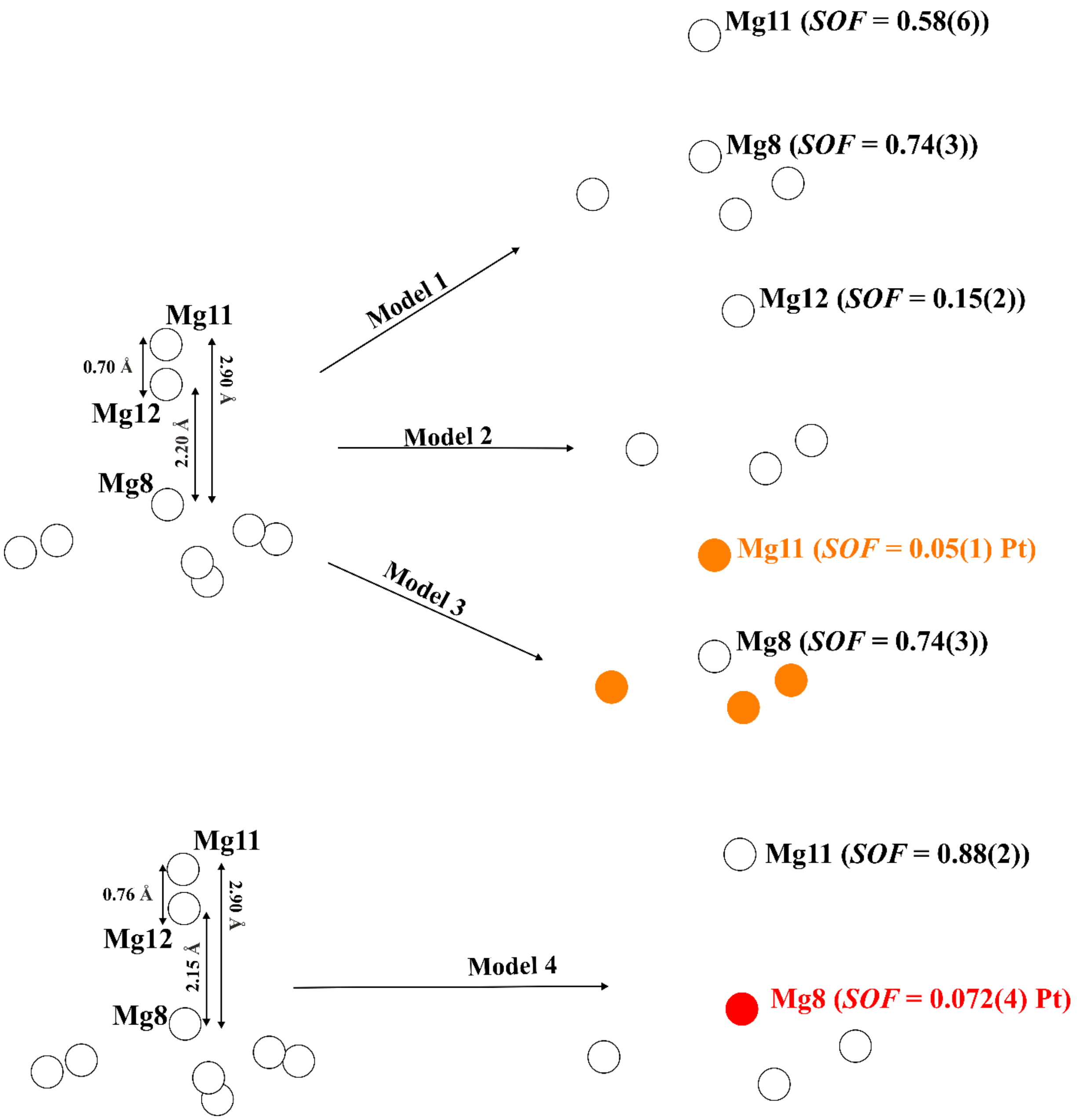

Figure 8S. (top and bottom left) highlighted distances in the tetrahedron between the different sites affected by disorder, according to the two different possibilities to solve the crystal structure (see Tables 2, 4S, 5S and 6S). (right) From the top to the bottom are represented the 4 different scenarios according to model1, model2, model 3 and model 4. Color code: orange – Pt at Mg11 position; red – Pt at Mg8 position; empty circle on the right – Mg at Mg8, Mg11 and Mg12 positions. In the crystal structure of $Mg_{29-x}Pt_{4+y}$ the sites which are influenced by disorder (Mg8, Mg11 and Mg12) are distributed between the vertices (Mg11 and Mg12) and in the center (Mg8) of a tetrahedron; the center coincides with the origin (0, 0, 0) of the crystallographic structure. Below a graphical description of the four possible models described in the main text is represented.

As already reported, a possibility to solve the crystal structure is to consider a mixed occupation Mg/Pt for Mg11 and a partial occupation of the Mg8 and Mg12 by Mg. In this situation, an analysis of the interatomic distances between the three sites reveals that if Mg12 is occupied the simultaneous occupation of Mg11 and Mg8 is not possible because of too short interatomic distances. This is in line with model 1, model 2 and model 3.

Another possibility to solve the structure, is to consider the presence of a platinum atom in 4*a* position (Tables 4S-6S). In this case, making again some considerations about interatomic distances between the three mentioned sites, we can obtain the model 4 by considering the center of the tetrahedron being occupied by 7.2% of Pt and vertices by 88% Mg11. If Mg12 is occupied, the simultaneous occupation of Mg11 and Mg8 is not possible due to too short interatomic distances, which would again lead to the situation presented in model 2.

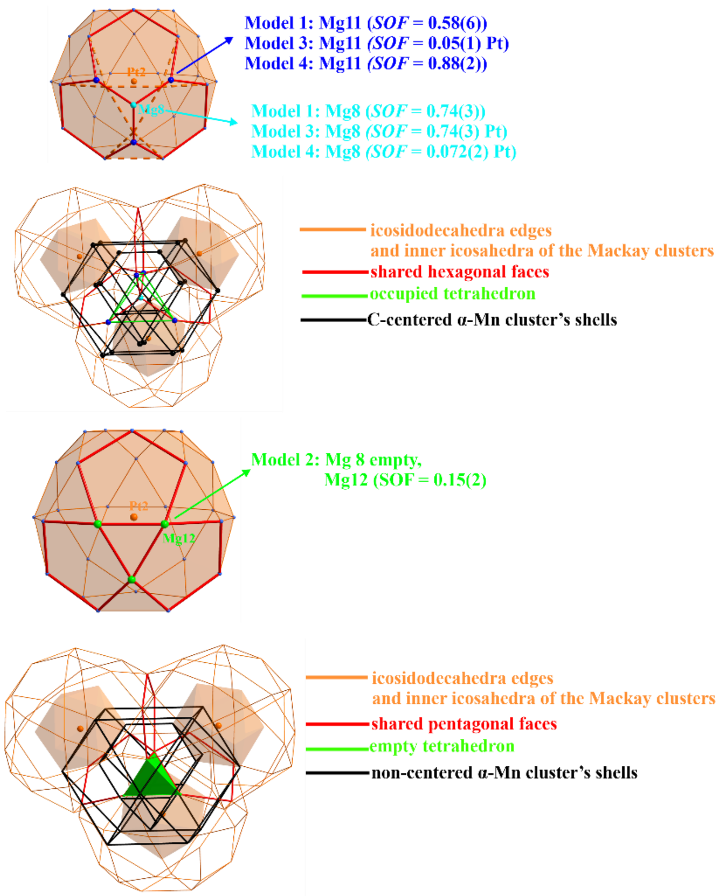

Figure 9S. Graphical representation of the Mackay cluster and α–Mn cluster according to the four different models, reported in the main text. (top) Not ideal icosidodecahedron of the Mackay cluster, according to the model 1, model 3 and model 4. (middle-top) Three icosidodecahedra of the Mackay cluster at distance 8.56 Å, sharing the hexagonal faces and the center of the tetrahedron (turquoise); in black are highlighted the concentric shells of the C-centered α–Mn cluster. (middle-bottom) Ideal icosidodecahedron of the Mackay cluster, according to the model 2. (bottom) Three icosidodecahedra of the Mackay cluster at distance of 8.56 Å, sharing the pentagonal faces and the empty green tetrahedron at the center. In black are highlighted the concentric shell of the non-centered α–Mn cluster. For simplicity only three of the four icosidodecahedra at distance 8.56 Å are shown.

It is important to understand how the three sites affected by the disorder are distributed in the crystal structure, since they belong to two important building blocks of the cubic structure, which are the second shell of the Mackay cluster (icosidodecahedron) and the α–Mn cluster. In the case of model 1, model 3 and model 4 the icosidodecahedron of the Mackay cluster is not perfect because the three pentagonal faces and one triangular face are substituted by three hexagonal faces, for a total of 31 atoms per shell. Moreover, the icosidodecahedra at distance 8.56 Å are sharing the hexagonal faces and the Mg8 at the center. With regard to the α–Mn cluster, a total of 29 atoms in the cluster can be count, with the center (C) of the cluster being occupied (top and middle-top, Figure 9S). In the case of model 2, the icosidodecahedron of the Mackay cluster shows an ideal shell with pentagonal and triangular faces which are sharing edges and vertices for a total of 30 atoms. As a consequence of the absence of the Mg8, the four icosidodecahedra at distance 8.56 Å, are sharing an empty tetrahedron with Mg12 at the vertices, and the α–Mn cluster becomes a non–centered α–Mn unit with 28 atoms in the unit (middle-bottom and bottom, Figure 9S).
